# Fluorometric Measurement of Individual Stomata Activity and Transpiration *via* a “Brush-on”, Water-Responsive Polymer

**DOI:** 10.1038/srep32394

**Published:** 2016-08-31

**Authors:** Minjeong Seo, Dong-Hoon Park, Chan Woo Lee, Justyn Jaworski, Jong-Man Kim

**Affiliations:** 1 Department of Chemical Engineering, Hanyang University, Seoul 04763, Korea; 2 Institute of Nano Science and Technology, Hanyang University, Seoul 04763, Korea

## Abstract

Much of atmospheric water originates from transpiration, the process by which plants release H_2_O from pores, known as stomata, that simultaneously intake CO_2_ for photosynthesis. Controlling stomatal aperture can regulate the extent of water transport in response to dynamic environmental factors including osmotic stress, temperature, light, and wind. While larger leaf regions are often examined, the extent of water vapor release from individual stomata remains unexplored. Using a “brush-on” sensing material, we can now assess transpiration using a water-responsive, polydiacetylene-based coating on the leaves surfaces. By eliciting a fluorometric signal to passing water vapor, we obtained information regarding the activity of individual stomata. In this demonstration, our results prove that this coating can identify the proportion of active stomata and the extent of transpirational diffusion of water in response to different conditions.

Common to all plants is the need to accommodate the inward diffusion of CO_2_ while controlling the outward flux of water. Pairs of guard cells distributed on plant leaves control the aperture of stomatal pores and have become the means for regulating such water loss while facilitating the plants demand for carbon dioxide by serving as an effective gating component^1^. Plant adaptation and health in response to environment perturbations, including water status, temperature, and light intensity among others factors, have been assessed in the past by measuring the stomata or determining the diffusion conductance from the surface of leaves^2^. Existing models for stomatal conductance of water vapor from leaves have already been thoroughly reviewed^3^, where it has been noted that a complete view of stomatal regulation is necessary to gain a more fundamental understanding of guard cell functioning.

It is well known that the rate of water movement into the plant is dictated in large part by the soil water potential as well as the ease of water passage through the soil, which are partially determined by the size of the soil particles and water availability^4^. In contrast, water movement out of the plant occurs by stomatal transpiration, which occurs upon opening of stomata present on the leaves. This opening of stomatal pores facilitates transport of water out of the plant as water vapor released into the atmosphere resulting in a negative hydrostatic pressure within the plant. The water potential and hydrostatic pressure within the plant are thus directly related to transpiration by the concentration gradient of water vapor at the leaf surface. High rates of transpiration are generally believed to occur when the stomata remain maximally open, wherein the water that has been taken up into the plant is quickly released into the atmosphere such that the xlyem will not possess a positive pressure^5^. Should such rates of transpiration remain high even when water movement into the root system is slow (i.e., drought) then wilting will inevitably occur unless dynamic opening of the stomatal pore can help the plant meet its demands for carbon fixation while trying to avoid dehydration. Natural anti-transpirants, such as the metabolic inhibitor abscisic acid (ABA), which can induce stomatal closure, or synthetic film forming chemicals can prevent excessive water loss and have found a myriad of agricultural uses to improve the productivity and quality of crops^6^,^7^. For example, certain crops with excessive water requirements due to high rates of water loss *via* transpiration may be grown under more arid conditions if supplied with anti-transpirants^8^,^9^. It is clear that a complete understanding of the stomatal response remains critical to understanding and controlling transpiration in plants for the purpose of water use efficiency in agriculture^10^.

While the average rate of transpiration can depend on a plants water requirements and extent of photosynthesis based on genetic adaptation, it can also be effected instantaneously by a number of external factor such as light, humidity, and wind^11^. Examining the extent of transpiration is crucial for determining plant adaptation to environmental stresses. It is often examined by measuring the diameter of the stomata as well as their frequency or alternatively by looking at the bulk conductance of leaves; however, many combinations of diameter and number density may result in equivalent levels of stomatal conductance of water vapor^12^. Despite this, past techniques including microscopy, the formation and analysis of silicone rubber leaf impressions, and scanning electron microscopy (SEM) have been used to assess stomatal size and frequency^13^,^14^. In addition to being time consuming, traditional stomata counting can be subject to variability due to complex morphological features at the leaf surface. In the following work, we reveal a fluorescent sensor based scheme for assessing transpiration by the examining the relative release of water vapor from individual stomata as well as providing a means for assessing the stomatal distribution. We have observed heterogeneous levels of transpiration activity across the surface of leaves, which warrants future studies to determine if this is an effect of the stomatal aperture or other possible causes as mentioned below. A breakthrough of this fluorometric measurement technique is the capability of examining the conductance of water vapor from individual stomata rather than the bulk conductance as is typically done with a conventional leaf porometer^15^,^16^. In addition to the possibility of differences in aperture diameter, the heterogeneity in activity across the leaf surface may also be a direct result of variance in the internal water vapor concentration in the air spaces beneath the stomata. This may be effected by unequal proximity of individual stomata to the xylem or perhaps differences in the distribution and density of nearby mesophyll cells from which the water vapor is derived.

In our system (Fig. 1) we utilize a “brush-on” water responsive polymer that serves the function of a fluorescence filter capable of detecting the presence of water derived from stomatal transpiration. In comparison to bulk conductance measurements, this “brush-on” transpiration sensor may also prove to be a valuable field research tool for evaluating transpiration; however, with this sensor, assessment can be made at the level of individual stomata and the sensor may also discern the proportion of active stomata *vs.* inactive stomata at the whole leaf scale, not previously possible with conventional techniques. The water responsive polymer coating employed is a polydiacetylene (PDA) derivative. Polydiacetylene^17^,^18^,^19^,^20^,^21^,^22^,^23^,^24^,^25^,^26^,^27^,^28^,^29^,^30^,^31^,^32^,^33^,^34^,^35^,^36^,^37^,^38^,^39^,^40^,^41^,^42^,^43^,^44^ based materials have long been known to polymerize into what is referred to as a blue, non-fluorescent state, or if triggered by the presence of a particular stimuli they may exhibit a red, fluorescent state. The configurations of the red and blue phase PDA differ by their extent of pi-orbital overlap of the backbone as determined by alignment and stabilization of their side-chains. Once coated on a surface and polymerized, we find that the polydiacetylene (PDA) in its blue phase may act as a filter with light absorption up to nearly 690 nm that overlaps the absorption maxima of chlorophyll a and chlorophyll b at 663 nm and 647 nm, respectively^45^, thereby reducing the native chlorophyll fluorescence emission band (665–690 nm)^46^. However, in locations in which the stomata are active in transpiration, the blue phase PDA is locally converted to the red phase PDA due to molecular interactions with water. A resulting shift in the absorption spectra allows for the underlying chlorophyll fluorescence as well as the red phase PDA’s inherent fluorescence signal to be observed. Depending on the extent of water conductance at the stomata, the fluorescence intensity was found to vary revealing that the abaxial surface of the leaf has significant heterogeneity in the activity of individual stomata. Herein we provide details of this unique approach to determining the proportion of active stomata, which by traditional techniques was previously unidentifiable at the level of individual stomata.

## Results

### Water-responsive polydiacetylene

**Figure 1:**
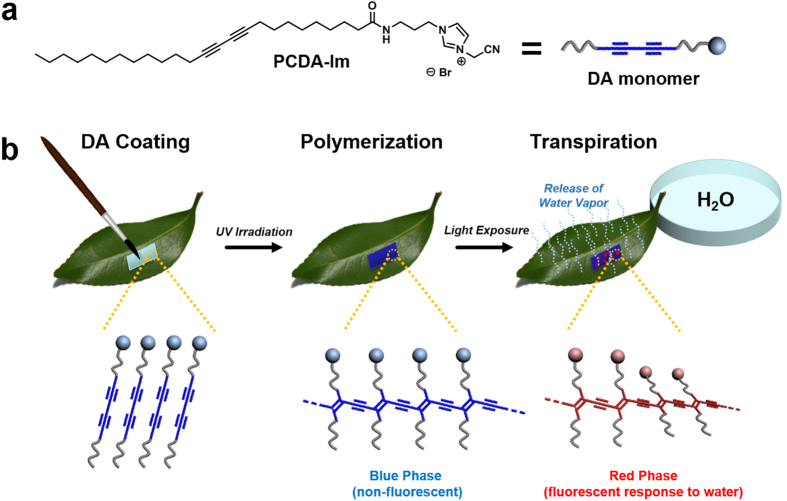
Schematic of PDA sensor, coating process, and analysis of transpiration. (**a**) Structure of PCDA-Im monomer used for polymer coating. (**b**) Procedure for coating PCDA-Im monomers onto the under-side (abaxial surface) of a leaf followed by UV polymerization to afford the non-fluorescent “blue phase” PDA and subsequent exposure of the petiole to water to permit transpiration. Conductance of water vapor at the site of active stomata causes a localized “red phase” response in the PDA sensor coating.

Previously, we have developed a Cs ion containing PDA film as a hygroscopic, water-responsive polymer for fingerprint analysis by mapping of the human sweat pores after touching the PDA-Cs film surface^47^. In a more recent effort, the hydrochromic PDA system was further optimized, and the PDA derived from PCDA-Im (Fig. 1a) afforded both increased stability in ambient air conditions and inkjet compatibility^48^. Strong intermolecular interactions among the PCDA-Im monomers allow close, constrained packing which facilitates significant pi-orbital overlap upon UV polymerization of the PDA backbone providing the blue phase sensor. The presence of water molecules would immediately result in ion-dipole interactions with the hygroscopic imidazolium head-group of the PCDA-Im thereby causing distortion in the backbone due to rearrangement of the side-chains. With a related hydrochromic response in mind, a “brush-on” strategy for coating leaf surfaces with Br ion containing PCDA-Im was developed, such that the extent of water vapor released from the leaves could be assessed by a fluorescent response. From this we examined the ability of the blue phase polymerized PCDA-Im coated on the bottom surface of leaves to elicit a change to red phase PDA to provide a fluorescent signal in response to transpiration of water from individual stomata. Specifically, we find that the polymerized PCDA-Im exhibited a predictable change in the absorption as well as fluorescence properties upon exposure to water due to its hydrochromic phenomenon. Based on our investigations with exposure of PCDA-Im directly to aqueous solutions containing weak acid or salt showing no difference in the signal to water, we need not be concerned about any interfering effects due to the presence of plant chemicals such as abscisic acid or KCl.

### Polydiacetylene coating as a fluorescence filter

**Figure 2:**
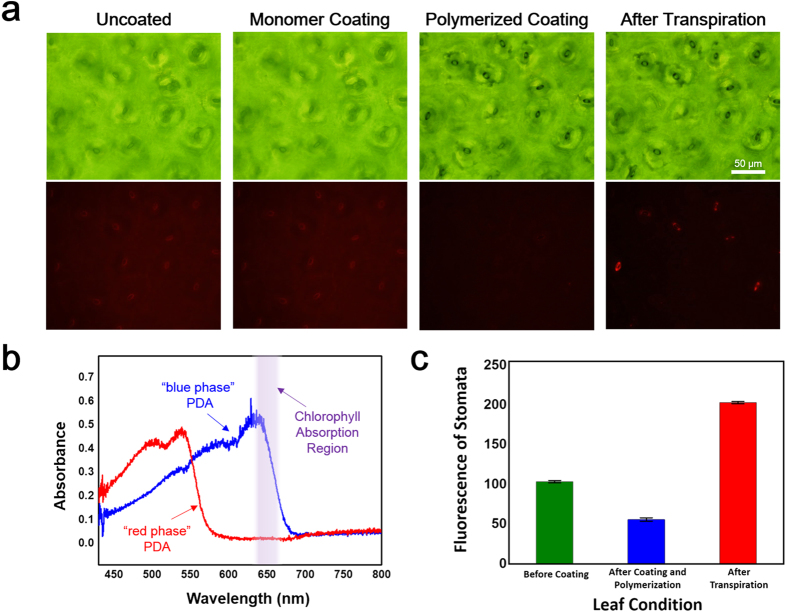
Fluorescent response of PDA coating to stomatal water vapor from transpiration. (**a**) Optical (top) and fluorescence (bottom) microscopy images of the under-side (abaxial surface) of a *Camellia japonica* leaf before coating, after monomer coating (pre-polymerization), after polymerization of PDA monomers, and after transpiration. (**b**) Absorption spectra of blue phase and red phase PDA coatings. Once the PDA coating has been polymerized on the leaf the blue phase PDA (which absorbs up to ~690 nm and has no fluorescence) is found to act as a filter as seen in the fluorescence images, restricting the native red fluorescence arising from chlorophyll a and b due to overlap of the PDA absorption spectra in this region. In contrast the PDA coating above the active stomata is exposed to water vapor as a result of transpiration thereby triggering the red phase of PDA (which absorbs below ~550 nm and has a red fluorescence). The induction of a PDA fluorescence signal in response to water vapor along with the shifting of the PDA absorption spectra as to no longer overlap that of chlorophyll results in a detectable red fluorescence localized above the stomata wherein the intensity is a function of the stomatal activity (*i.e*, the amount of transpired water). (**c**) Comparison in the stomatal fluorescence prior to PDA coating (green bar) shows the fluorescence contribution from chlorophyll, while after PDA coating polymerization the fluorescence from the stomata is filtered by approximately 50% (blue bar). Intense fluorescence is observed for active stomata after transpiration (red bar).

From Fig. 2a, we can observe that the optical and fluorescence images of the leaf are almost identical before and after coating the monomeric PCDA-Im. It is worth noting that a background red fluorescence can be observed arising from the leaf surface for the uncoated leaf as well as the monomer coated leaf prior to polymerization. This intrinsic fluorescence is due to chlorophyll emission at ~690 nm^49^. In contrast, after UV polymerization of the PCDA-Im coating, we find that the intrinsic fluorescence is absent as a result of the polymerized PDA coating serving as an effective filter (Fig. 2a). Specifically, PDA in the reported “blue phase” has an absorption spectra that reaches up to ~690 nm (Fig. 2b) that may serve as a light filter thereby reducing absorption and hence fluorescence emission from chlorophyll a and chlorophyll b. Interestingly, after the induction of transpiration we find that the PDA coating displays a fluorescent “red phase” exactly at the location of the stomata from which water vapor is released during transpiration by the leaf (Fig. 2a). This fluorescence is believed to be attributed to a combination of the inherent red fluorescence of the red phase PDA as well as the natural chlorophyll fluorescence, since the shift in the absorption spectra for the red phase PDA no longer acts as a filter for light in this wavelength range (Fig. 2b). The reduced fluorescence intensity after polymerization and subsequent increase of the fluorescence intensity of stomata after transpiration is displayed in Fig. 2c.

### Response of hydrochromic polymer to water vapor from stomata

**Figure 3:**
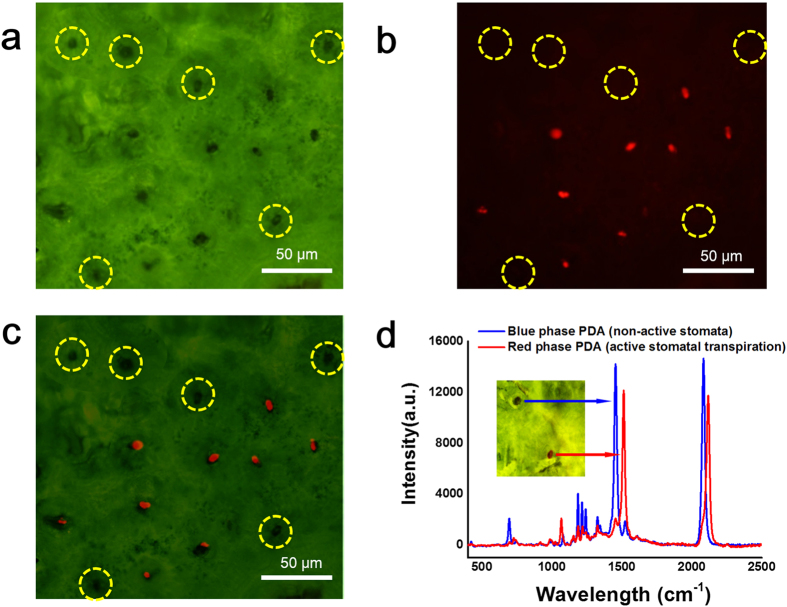
Examining heterogeneity in water vapor release from stomata and assessing the effect on PDA structure. (**a**) Optical image, (**b**) fluorescence image, and (**c**) merged image of PDA coated leaf showing stomata with different levels of transpiration. Several stomata secreted no detectable levels of water vapor as indicate by the dashed yellow circles. (**d**) Comparison of Raman spectra of the PDA coating above an active stomata, in which a fluorescent red phase PDA was formed due to water vapor from transpiration, relative to an inactive stomata, in which the PDA coating remained a non-fluorescent blue phase. The presence of water vapor from transpiration caused the PDA coating over only the active stomata to adopt a different configuration resulting in the fluorescent signal.

**Figure 4:**
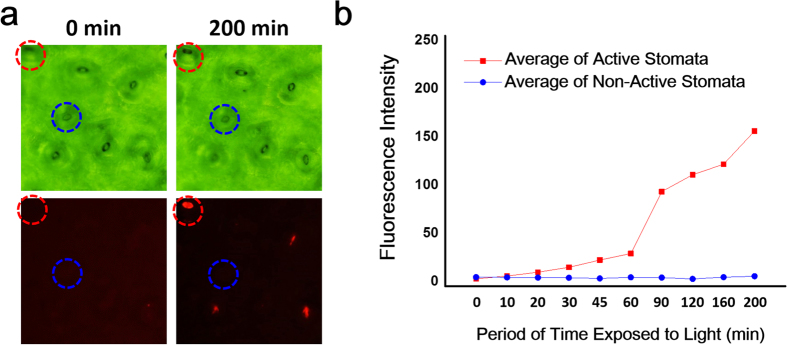
Assessing the time dependent release of water vapor from stomata during transpiration. (**a**) Optical (top) and fluorescence (bottom) images of active (red circle) and non-active (blue circle) stomata. (**b**) Average intensity of red fluorescence as a function of time after exposing leaf petiole to water for transpiration.

We also observed the leaf surface directly by SEM (Supplementary Figure 1) to find the occurrence of both open and closed stomata. In order to prove the fluorescent spots displayed in Fig. 2a originate from the PDA above the active stomata, the optical (Fig. 3a) and fluorescence (Fig. 3b) microscopy images from a leaf after transpiration, were superimposed (Fig. 3c). Close inspection of the superimposed image shows that the fluorescent spots are located only at the stomata. Interestingly, we also found some stomata sites where their apparent transpiration activity was negligible (Fig. 3c, dashed yellow circles). Examining the PDA coating at the location of individual stomata we found that for the case of the red phase PDA above active stomata where there was sufficient water vapor release, the conjugated polymer system was perturbed. Specifically, the Raman spectra of the red-phase PDA present over the active stomata exhibited a shift in the alkyne (C≡C) and alkene (C=C) bands^50^ to higher frequencies as compared to the blue-phase PDA present over locations of inactive stomata (Fig. 3d). Interestingly, only at one of the stomata was there a sufficient amount of water vapor released to induce a detectable change in the PDA configuration. Through these results, we provide evidence of heterogeneity in the conductance of water vapor from stomata across the leaf surface, but the exact cause for the heterogeneity remains to be determined in future studies. Further examining our approach, we observed the amount of time necessary for the leaf to exhibit transpiration activity. Specifically, we assessed different light exposure times while analyzing the extent of transpiration by the PDA’s fluorescence response to water vapor (Fig. 4). As presented in Fig. 4a, before exposure to water, the originally non-fluorescent stomata (dashed red circle) emits a bright red fluorescence only after transpiration when observed at 200 min from the time of providing the leaf with water. The fluorescence intensity profile (Fig. 4b) proves that the active stomata displays a time-dependent increase in the fluorescence intensity, while the inactive stomata shows almost no variation in the fluorescence intensity.

### Environmental influence on the proportion of active stomata

**Figure 5:**
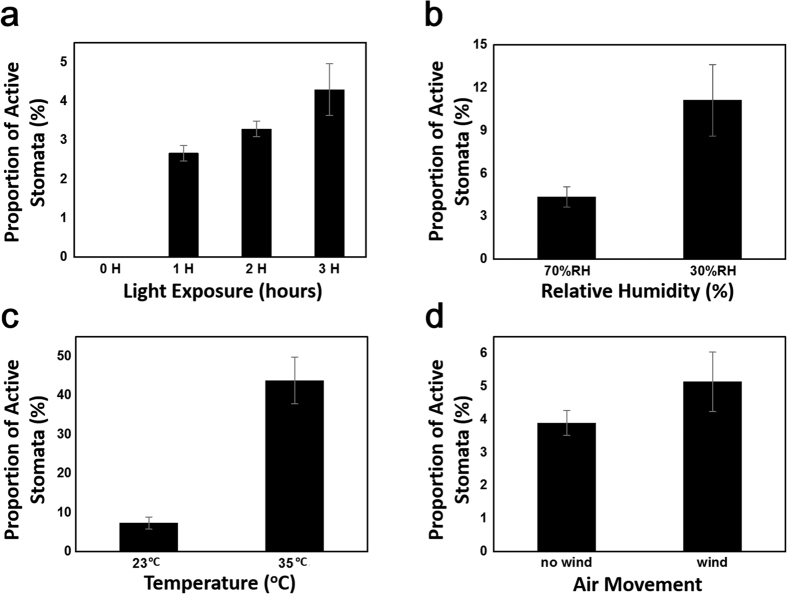
Determining environmental influence on the proportion of active stomata using PDA coating. (**a**) Light duration, (**b**) humidity, (**c**) temperature, and (**d**) wind were used as variable environmental conditions to assess the effect on transpirational diffusion of water from stomata on the surface of leaves. The proportion of active stomata was determined by the fluorescence signal generated on the PDA leaf coating as its response to moisture released from the stomata by transpiration.

To explore the potential of this coating material as a means for assessing environmental effects on transpiration at the level of individual stomata, we examined the stomatal activity in response to variations in light, humidity, wind, and temperature. From Fig. 5a, we see that with no light the stomatal activity was negligible as no fluorescent due to transpiration of water could be observed. After one hour however, an increase in transpiration activity to low levels was observed as indicated by the relatively higher number of stomata possessing low intensity fluorescence. After two hours, the activity increased significantly and by three hours there was a large number of stomata that were highly active as determined by the intense fluorescence due to transpiration. Next we examined the humidity effect since the difference in water vapor concentration between the external ambient air relative to the internal leaf airspace underlying the stomata is one of the key forces guiding transpiration. Indeed, as seen in Fig. 5b, the higher levels of humidity were found to result in less transpiration from the stomata even when under illumination. In the same manner, when exploring different leaf temperatures we found that the extent of stomatal activity was significantly diminished at lower temperatures (Fig. 5c). The effect of leaf temperature on transpiration occurred to a much greater extent than the humidity; however, this was to be expected as the vapor pressure of water increases exponentially with temperature. This is believed to have occurred as the leaf itself was heated rather than the surrounding bulk air. The leaf itself was heated in this case purposefully, since if the bulk ambient air were heated we would have expected an additional variable of the saturated water vapor content and hence the humidity to have also changed within the same experiment. Finally, examination of the effect of wind on stomatal activity revealed an increase in transpiration in the case of moving air (Fig. 5d). This was anticipated to play such a role on transpiration as wind can disrupt the still air surrounding the stomatal aperture, thereby decreasing what is referred to as the leaf boundary layer resistance to transpiration. If the boundary layer of still air is thick, it can result in a high resistance to transpirational water loss^51^. While leaves cannot control this boundary layer in real time, the shape and in some cases the movement of the leaves can play a role in altering this resistance to transpiration. On a faster time scale, control over the stomatal aperture is known to be initiated by plants to control the diffusion of water through the pore as regulated by its aperture diameter (*i.e.*, the leaf stomatal resistance)^5^. The ability of the stomata aperture to regulate transpirational flux of water from the leaf surface has been noted to have less effect in still air due to the high boundary layer resistance, while moving air breaks down the boundary layer to allow a much greater change in transpirational flux to be achieved for a given change in stomatal aperture^51^,^52^.

### Differences in the extent of transpiration from individual stomata

**Figure 6:**
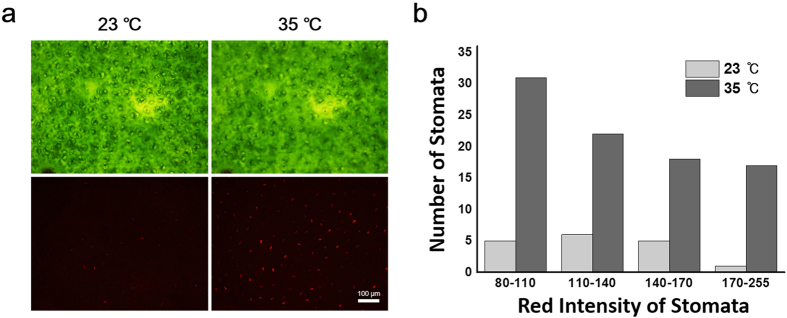
Examining the difference in transpiration from individual stomata across the leaf surface. The relative extent of transpirational diffusion of water from individual stomata can also be determined by the red intensity from (**a**) fluorescence images under different environmental conditions. In addition to using PDA coating to assess the surface distribution of active stomata, the fluorescence response can also be used to construct (**b**) histograms to compare the relative extent of transcription among all active stomata.

In a specific case in which the leaf is first exposed to a lower temperature followed by a higher temperature to elicit increased diffusion of water (Fig. 6a), we see that each individual stomata can be observed to have distinct levels of activity. The distribution of the fluorescent intensity due to transpiration can be observed at the stomatal level or a comparative histogram can be used to reveal the differences in the extent of transpiration of the active stomata (Fig. 6b). In looking back at the effect of wind, this difference in activity from stomata to stomata may be a factor of local heterogeneities along the leaf surface in terms of air movement and relative thicknesses of the boundary layer of still air through which water vapor needs to diffuse. To confirm that this was not a factor of heterogeneity in the PDA film, we exposed the PDA coated leaf to a uniform amount of water vapor across the entire surface. From this we find that the PDA at the location of stomata previously determined inactive by our assay (non-fluorescent) were now, after exposure to water vapor, triggered into the red fluorescent state (Supplementary Figure 2). This confirms that the PDA coating at the location of the inactive stomata was indeed effective in being able to give a red response but that it did not initially appear red due to a lack of transpiration from those particular inactive stomata. Across the leaf surface, we confirmed that the saturating water vapor triggered a uniform response across the leaf due to the uniform PDA coating. As mentioned previously, we find a consistently higher red fluorescence intensity at the site of the stomata due to the inherent red fluorescence of chlorophyll^49^ present in the guard cells surrounding the pore. Furthermore, we have shown that this “brush-on” coating based sensor may be used for a variety of leaves (Supplementary Figure 3) in which we also observed heterogeneity in the extent of transpiration across the surface of the leaves.

## Discussion

In our assessment, we have explained how several environmental factors can influence transpiration by physical mechanisms including changes in diffusion resistance and water vapor pressure. Nonetheless, it is important to note that the guard cells, which comprise the stomatal complex and regulate the effective pore diameter, are already well known to be responsive to environmental stimuli such as light and temperature. A significant amount of work has been done throughout the scientific community over the years to provide insight into biochemical mechanisms by which such factors effect stomatal aperture^53^,^54^,^55^,^56^,^57^. In our work, we hope to contribute to existing models by our observations of transpirational water flux at the level of individual stomata. In addition, we see that there is heterogeneity in stomatal activity when observed across the leaf surface. The exact source of the heterogeneity remains unclear and warrants future studies to reveal if this correlates with the aperture of the stomata. The possibility of local variations in air movement due to leaf shape or perhaps a collapse of certain xylem conduits for water transport^58^ within the leaf may also be viable causes. Similarly, differences in the distance between individual stomata and nearby xylem conduits may also be considered, as this might result in varying water vapor concentrations in the internal air spaces of different sub-stomatal cavities (i.e., the open space beneath the guard cells of individual stomata). By providing this fluorometric approach for observing transpiration from individual stomata, we hope to offer this scheme as a valuable addition to field experiments as the sensor coating may easily be applied to the leaf surface prior to excision from the plant. As such, this coating approach may give a more accurate means for determining the stomatal behavior as compared to methods that require acquisition or excision of the leaf prior to the analysis, since this has been known to influence the rates of transpiration^59^,^60^,^61^.

In summary, we have shown that the hydrochromic polymer of PCDA-Im may provide an effective coating on the abaxial (under-side) surface of leaves for evaluating the extent of transpirational water loss from individual stomata. Owing to the overlapping absorption spectra of PDA and chlorophyll, we see that the PDA coating acts as a fluorescence filter in the blue phase, which upon exposure to moisture during transpiration shifts to a red phase allowing the fluorescence of the PDA and underlying chlorophyll to then be observed. With this approach we may assess the transpiration from discrete stomata and also provide a precise mapping of the distribution of stomatal activity on the leaf surface. Foreseeably, the observations made in this work may impact the way in which we interpret current studies of stomatal distribution and morphology, and we expect this will only strengthen the foundation on which we use transpiration to assess plant productivity and health with respect to internal as well as environmental stress^62^,^63^.

## Methods

### Synthesis of [3-(Cyanomethyl)-1-(3-(pentacosa-10,12-diynamido)propyl)-1H-imidazol-3-ium bromide] (PCDA-Im)

The PCDA-Im was prepared as previously described in literature^48^. In brief, a 250 mL round bottom flask, 693 mg (2 mmol) of 10,12-pentacosadiynoic acid (PCDA, purchased from GFS Chemicals) was combined with 20 mL of methylene chloride and to it was added 345 mg (3 mmol) of N-hydroxysuccinimide (NHS) followed by 766 mg (4 mmol) of 1-ethyl-3-(3-dimethylaminopropyl)carbodiimide (EDC). The reaction was stirred under nitrogen overnight at room temperature followed by extraction in ethyl acetate and evaporation overnight *in vacuo* to yield a white solid PCDA-NHS. Next, 887 mg of PCDA-NHS was combined with 0.35 mL (1.5 equiv.) of 1-(2-aminopropyl)imidazole (Sigma Aldrich) and 0.55 mL (2 equiv.) of triethyl amine in a 250 mL round bottom flask containing 20 mL of methylene chloride. The reaction was stirred overnight at room temperature followed by aqueous extraction. The sample was then added to a silica column using a 96:4 methylene chloride to methanol solvent system. The product PCDA-APrI, [N-(3-(1H-imidazol-1-yl) propyl) pentacosa-10,12-diynamide)], was concentrated *in vacuo*. 2 mmol of PCDA-APrI was dissolved in 20 mL of acetonitrile and to it was added 3 mmol of bromoacetonitrile (Sigma Aldrich) followed by reflux at 80 °C overnight. The sample was then concentrated *in vacuo* followed by precipitation and washing in cold hexane to provide the final product of [3-(cyanomethyl)-1-(3-(pentacosa-10,12-diynamido)propyl)-1H-imidazol-3-ium bromide], PCDA-Im.

### Acquiring and handling of leaf samples, coating, and polymerization

For examining the stomatal activity, leaves from the *Camellia japonica* plant were removed by hand at the node. Additional leaf samples were also tested as part of the proof of concept and the particular species are indicated on the corresponding figures. The bottom surface of the leaves were wiped clean to remove any dust using a tissue moistened with ethanol. The petiole of the leaves was cut with a razor and the freshly cut end of the leaf was placed in a 35 mm petri dish filled with deionized water. The dish was covered with parafilm, to reduce evaporation of the water, at which point the leaf and dish were placed in the dark for 12 hours. Using a synthetic fiber brush (HwaHong 848 Series Size 10 Oil Brush), 2 wt% of PCDA-Im (0.303 g PCDA-Im in 10 mL of chloroform) was brushed onto the bottom of the leaf with two strokes of the brush, as the bottom of leaves have a higher number of stomata. The chloroform immediately evaporated from the solution leaving behind the monomeric diacetylene (PCDA-Im); however, there was no noticeable trace of PCDA-Im on the surface when observed by optical or fluorescence microscopy, as the monomers are non-chromatic and non-fluorescent. Polymerization of PCDA-Im to afford the conjugated PDA film was performed on the surface of the leaves by exposure of the monomeric PCDA-Im film to a UV hand lamp (254 nm, 1 mW/cm^2^) for 20 seconds. The resulting polymerized film appeared with a slightly blue color; however, fluorescence microscopy revealed a more profound difference compared to the original leaf, namely a filtering of the fluorescence intensity due to the PDA film. After polymerization, the petiole of the leaves were placed back in the water of the petri dish for immediate use in examining the efficacy of the film in assessing transpiration.

### Inducing transpiration to assess the stomatal activity using the water-responsive PDA film

#### Effect of light

The top of the leaves were exposed to a broadband light source (35W Xenon lamp, 66140CBI from OSRAM XENARC Electronics). Optical and fluorescence microscopy was then used to observe each leaf at the same location in order to assess the number of stomata and fluorescence intensity from the individual stomata arising from transpiration. In order to first find the approximate time to expect a response in the PDA film to water vapor flux arising from transpiration, the same location on the leaf was imaged after 10, 20, 30, 45, 60, 90, 120, 160, and 200 minutes. An additional experiment, in which the same location on individual leaves were observed after 0, 1, 2, and 3 hrs of broadband light exposure, was conducted in order to identify the extent of transpiration at the level of individual stomata by observing the PDA film using optical and fluorescence microscopy. In each case, the experiment was repeated for 3 samples.

#### Effect of humidity

To examine the effects of humidity on the extent of transpiration of individual stomata, the topside of the leaf was exposed to a broadband light source as described above; however, in this case the system was placed in a custom made acrylic humidity chamber in order to generate a 70% relative humidity environment for which to compare the extent of stomatal activity at ambient relative humidity of 30%. The samples were maintained at this humidity and light treatment for 1 hour followed by optical and fluorescence microscopy to examine the extent of transpiration *via* the PDA film coating.

#### Effect of temperature

Leaves were also exposed to different temperatures of 23 °C under ambient laboratory lighting followed by 35 °C. The leaves were kept at this temperature for 1 hour followed by optical and fluorescence imaging of the PDA film coating on the leaves.

#### Effect of wind

To assess the effect of wind on transpiration, the leaves were first exposed to the broadband light source for 3 hr and then either imaged immediately or were exposed to a fan generating wind at 2.6 m/s during continued light exposure for 1 hour. The optical and fluorescence images of these were also recorded in order to assess the extent of transpiration as indicated by the water-responsive PDA film of polymerized PCDA-Im.

### Spectroscopic and microscopic characterization

Examination of the optical and fluorescence images of the leaves was conducted using an Olympus (BX51 W/DP70) microscope with a U-MWG2 filter set (510–550 nm excitation, exposure time of 142 ms). UV-visible absorption spectra were recorded using a single beam Agilent 8453 UV–visible Spectrometer (Agilent Technologies). The Raman spectra of the PDA film at locations on the leaf for active and inactive stomata respectively were collected with a Raman microscope (Horiba Scientific, LabRAM HR Evolution) using a 785 nm excitation laser. The leaf sample was immobilized onto a glass slide using double sided tape. For scanning electron microscopy (SEM), the leaf samples were immobilized onto an SEM plate using carbon tape as well as silver paste, and the leaves were sputter coated with platinum prior to SEM. SEM of the stomata was conducted with a JEOL(JSM-6330F) FE-SEM.

### Statistical analysis of stomatal activity from fluorescence images

To identify the ratio of active stomata and the extent of stomatal activity in transpiration, optical and fluorescence images obtained were processed using imageJ software to provide counting of the stomata as regions of interest (ROI) and measuring of the red fluorescence intensity for the individual stomata. In detail, the background subtracted images were first converted to 8-bit greyscale. The ROI of the greyscale images were then selected by adjusting the threshold to highlight all of the stomata structures. The number, area, as well as the max and mean red intensity of the stomata were finally measured from the ROI.

## Additional Information

**How to cite this article**: Seo, M. *et al*. Fluorometric Measurement of Individual Stomata Activity and Transpiration *via* a “Brush-on”, Water-Responsive Polymer. *Sci. Rep.* **6**, 32394; doi: 10.1038/srep32394 (2016).

## Supplementary Material

Supplementary Information
