# Supplementary material for: Fluorometric Measurement of Individual Stomata Activity and Transpiration via a “Brush-on”, Water-Responsive Polymer
Source: Sci Rep. 2016 Aug 31;6:32394. doi: 10.1038/srep32394 (PMC5006157; doi:10.1038/srep32394)
Supplement: Supplementary Information [file srep32394-s1.pdf]

Supplementary Information

Fluorometric Measurement of Individual Stomata Activity and Transpiration *via* a "Brush-on", Water-Responsive Polymer

Minjeong Seo[1], Dong-Hoon Park[1], Chan Woo Lee[2*], Justyn Jaworski[1,2]*, Jong-Man Kim[1,2]*

[1]Department of Chemical Engineering, Hanyang University, Seoul 04763, Korea.

[2]Institute of Nano Science and Technology, Hanyang University, Seoul 04763, Korea

**J.-M. Kim: jmk@hanyang.ac.kr; J. Jaworski: justynj@hanyang.ac.kr; C. W. Lee: lcw@hanyang.ac.kr*

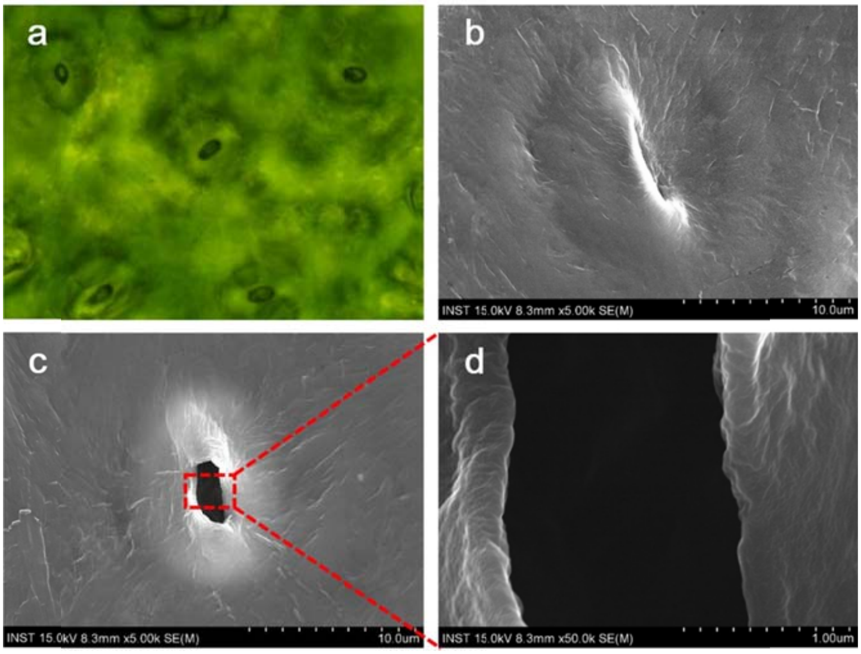

Supplementary Figure 1. Images of open and closed stomata. a) Optical image of the leaf surface after transpiration showing the stomata pore surrounded by guard cells. SEM images of b) closed stomata and c) open stomata with d) the higher magnification image showing a pore diameter of roughly 1.3 um and revealing the sub-stomatal cavity of open space beneath the guard cells from which water vapor is released during transpiration.

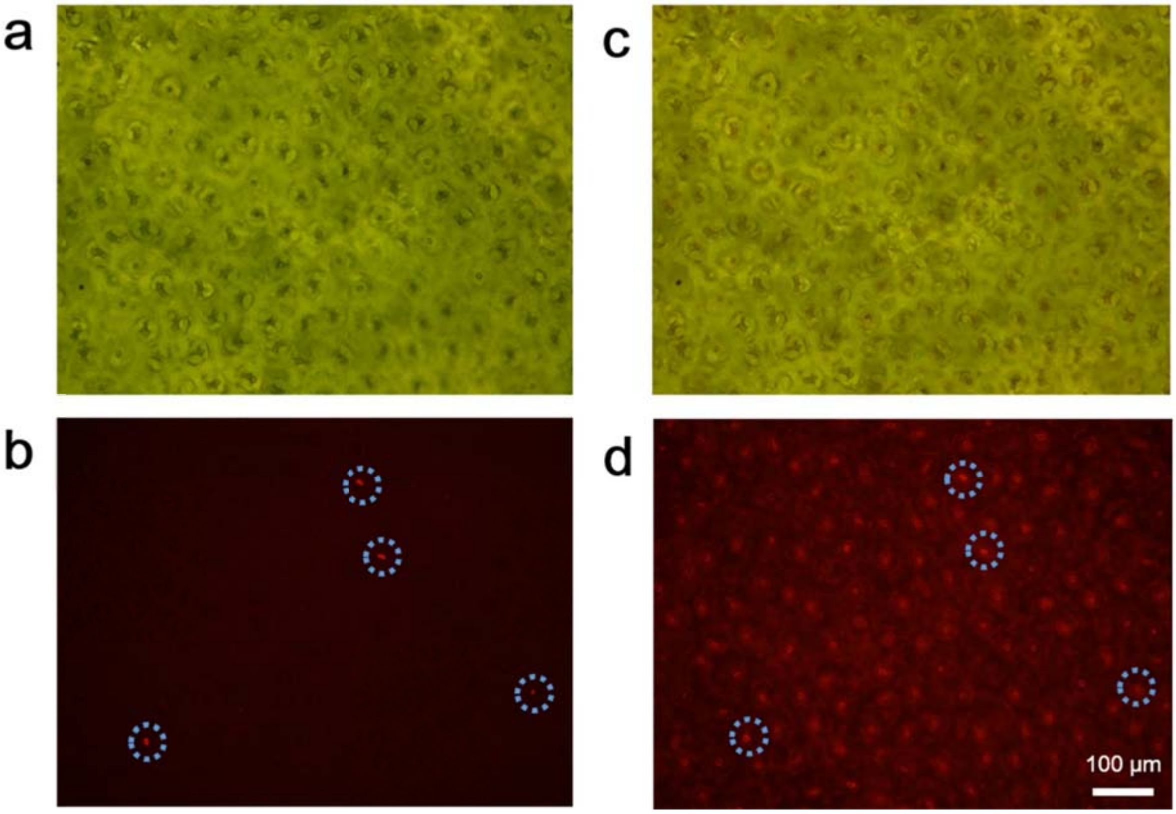

Supplementary Figure 2. a) Optical and b) fluorescence images of PDA coated leaf surface after transpiration with circles indicating the stomata with the highest transpirational activity in release of water. On the right side, the c) optical and d) fluorescence image of the same leaf is seen after exposure to saturated water vapor to induce the red phase PDA over the entire coating. The uniformity of the PDA coating is revealed in this fluorescent image wherein each of the stomata can be easily observed as a brighter intensity red spot due to the intrinsic chlorophyll fluorescence in addition to the red phase PDA fluorescence. Before exposure to saturated water vapor, the chlorophyll fluorescence was not observable due to the filtering effect of the PDA coating.

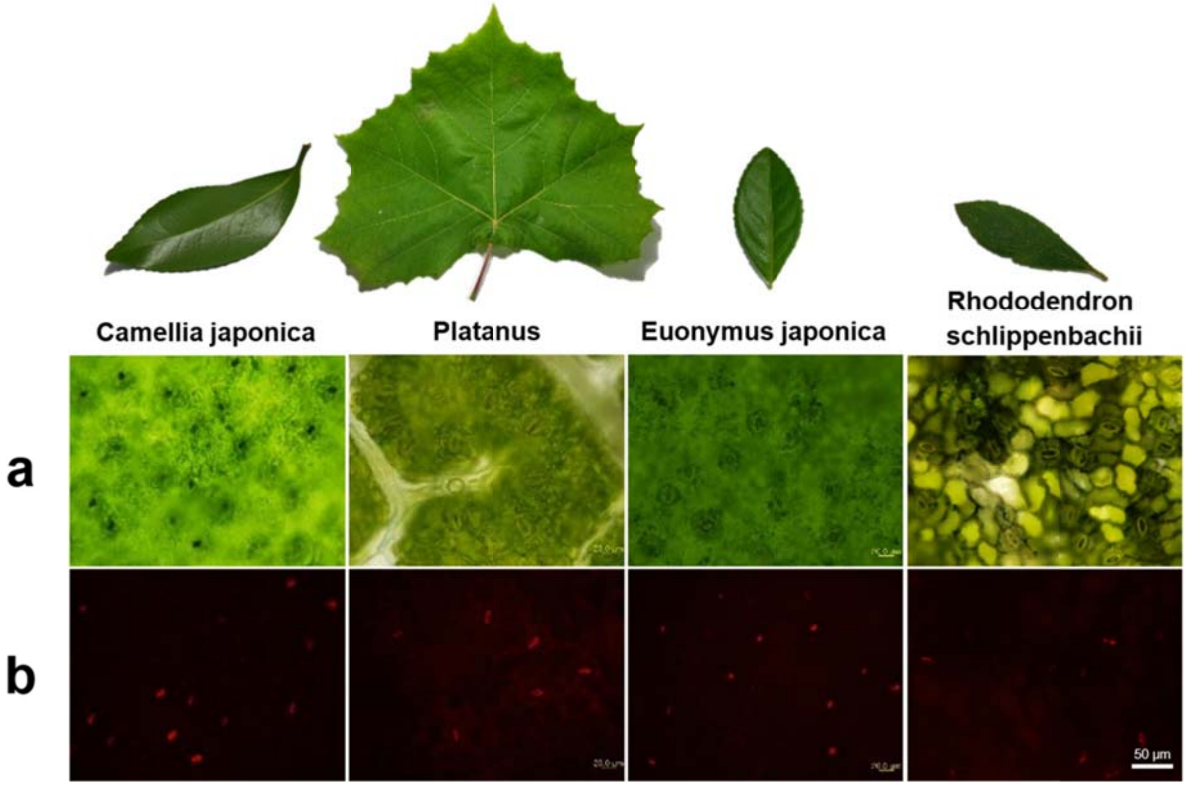

Supplementary Figure 3. Application of the PDA coating to leaves from various plant species including Camellia japonica, Platanus, Euonymus japonica, and Rhododendron schlippenbachii with the corresponding a) optical and b) fluorescence image after transpiration.
